# Actinobacterial diversity in limestone deposit sites in Hundung, Manipur (India) and their antimicrobial activities

**DOI:** 10.3389/fmicb.2015.00413

**Published:** 2015-05-05

**Authors:** Salam Nimaichand, Asem Mipeshwaree Devi, K. Tamreihao, Debananda S. Ningthoujam, Wen-Jun Li

**Affiliations:** ^1^Microbial Biotechnology Research Laboratory, Department of Biochemistry, Manipur UniversityCanchipur, Imphal, India; ^2^State Key Laboratory of Biocontrol and Guangdong Key Laboratory of Plant Resources, School of Life Sciences, Sun Yat-Sen UniversityGuangzhou, China; ^3^Molecular Genetics Laboratory, Department of Botany, North-Eastern Hill UniversityShillong, India; ^4^Yunnan Institute of Microbiology, Yunnan UniversityKunming, China

**Keywords:** actinobacterial diversity, limestone habitat, Hundung, antibacterial, biocontrol, biosynthetic genes, *Streptomyces*

## Abstract

Studies on actinobacterial diversity in limestone habitats are scarce. This paper reports profiling of actinobacteria isolated from Hundung limestone samples in Manipur, India using ARDRA as the molecular tool for preliminary classification. A total of 137 actinobacteria were clustered into 31 phylotypic groups based on the ARDRA pattern generated and representative of each group was subjected to 16S rRNA gene sequencing. Generic diversity of the limestone isolates consisted of *Streptomyces* (15 phylotypic groups)*, Micromonospora* (4)*, Amycolatopsis* (3)*, Arthrobacter* (3)*, Kitasatospora* (2), *Janibacter* (1)*, Nocardia* (1), *Pseudonocardia* (1) and *Rhodococcus* (1). Considering the antimicrobial potential of these actinobacteria, 19 showed antimicrobial activities against at least one of the bacterial and candidal test pathogens, while 45 exhibit biocontrol activities against at least one of the rice fungal pathogens. Out of the 137 actinobacterial isolates, 118 were found to have at least one of the three biosynthetic gene clusters (PKS-I, PKS-II, NRPS). The results indicate that 86% of the strains isolated from Hundung limestone deposit sites possessed biosynthetic gene clusters of which 40% exhibited antimicrobial activities. It can, therefore, be concluded that limestone habitat is a promising source for search of novel secondary metabolites.

## Introduction

Actinobacteria are major producers of secondary metabolites such as antimicrobial compounds, anticancer molecules and immunosuppressant agents (Takahashi and Omura, 2003). Since the beginning of antibiotic revolution, actinobacteria especially the genus *Streptomyces* have played major roles as antibiotic producers (Bérdy, 2005). However, the discovery of new antibiotics has not been in pace with the increase in demand for new antibiotics. The exhaustion of the usual terrestrial sources and the rise of resistant pathogens dictate the search for new antibiotics. To meet urgent clinical needs, screening for secondary metabolites from actinobacteria residing in unexplored habitats is warranted to, possibly, generate novel compounds.

Limestone habitats have high deposition of CaCO_3_ salts and may be considered a special habitat. Limited studies have been done for systematically exploring such habitats for novel actinobacterial strains (Kim et al., 1998; Groth et al., 1999a, 2001; Jurado et al., 2009; Nakaew et al., 2009; Niyomvong et al., 2012). Some reports are available on actinobacterial diversity in hypogean environments but the studies were focused on biodeterioration and conservation of paleolithic cave art. Actinobacteria implicated in deterioration of art work are considered serious risk factors if environmental changes promote their massive proliferation (Groth et al., 1999b; Portillo et al., 2009). To date, four new genera *Beutenbergia, Fodinibacter, Hoyosella*, and *Knoellia* have been reported from limestone habitats and related limestone ecosystems such as cave biofilms (Groth et al., 1999b, 2002; Jurado et al., 2009; Wang et al., 2009).

Manipur has a huge reserve of good quality limestone suitable for use in the manufacture of cement. The major limestone reserves have been located by Geological Survey of India near Ukhrul district, Manipur. Other limestone deposit sites include areas in Hundung, Phungyar, Meihring, Mova, Khonggoi, Lambui, and Paoyi. This paper reports the actinobacterial diversity profiling of the Hundung limestone deposit sites using ARDRA as the molecular tool for preliminary classification. ARDRA has been originally designed to decrease selection of duplicate clones in molecular analysis. It has been less frequently used in the study of bacterial diversity profiling unlike techniques such as DGGE. The paper also incorporates the results of antimicrobial screening of the Hundung actinobacterial strains.

## Materials and methods

### Sampling

Samples for the isolation of actinobacteria were collected from limestone deposit sites, Manipur, India (25.05°N, 94.33°E). The samples included limestones from the quarry site and rice field soil from the adjoining areas. These samples were aseptically packed in polyethylene bags and taken to the laboratory at the earliest possible time. Samples were then kept refrigerated till processing for isolation.

### Isolation of actinobacteria

Two synthetic media, Gauze's Medium No. 1 (GM1, pH 5.3) (Atlas, 1997) and Starch Casein Nitrate Agar (SCNA, pH 8.5) (Kűster and Williams, 1964), were used for the isolation of actinobacteria. Isolation was done using the procedure as described earlier (Nimaichand et al., 2012). The strains were preserved as lyophilized cultures and as glycerol suspension (20% w/v) at −80°C.

### Amplified ribosomal DNA restriction analysis (ARDRA; Heyndrickx et al., 1996)

Genomic DNA extraction and amplification of the 16S rRNA gene was done as described by Li et al. (2007). The amplified products were checked and purified by HiPurA™ 96 PCR product purification kit (HiMedia, India). Restriction digestion of the amplified 16S rRNA gene product was done using the enzymes *Hha*I and *Hin*fI (New England Biolabs, UK). The reaction mixture containing 10 μl amplified 16S rRNA gene product, 2 μl NEB buffer 4 (10X), 1 μl restriction enzyme (10 U/μl) and 7 μl deionized water was incubated at 37°C for 2 h and inactivated by heating at 70°C for 10 min. To 20 μl of the restriction digest, 4 μl loading dye (6X) (Promega) was added. Each sample was loaded in a well in agarose gel (3%, w/v) and the gel was run at 100 V for 90 min. In another well, 1 μl DNA ladder (100 bp) (Promega) was loaded to estimate the size of the restriction fragment. The gel was visualized in a gel documentation system (BIORAD Gel Doc EZ Imager). Bands between 100 and 1000 bp were used as reference points and banding patterns were analyzed by scoring the prominent bands. ARDRA band profiles for all the strains were scored with the help of GelBuddy software (Zerr and Henikoff, 2005) for the presence or absence of restriction fragments. A dendrogram was generated using the software package NTSYSpc version 2.02. The phylogenetic relationship was determined according to the method of unweighted pair group method with arithmetic mean (UPGMA; Sneath and Sokal, 1973). Based on the similarity indices (70% and above) in the dendrogram, all the strains were clustered into different phylotypic groups.

### Sequencing of 16S rRNA genes

Sequencing of a randomly-selected representative strain for each phylotypic group was done. The partial 16S rRNA gene sequence of the strain was identified using the EzTaxon-e server database (Kim et al., 2012). The phylogenetic tree of these strains based on neighbor-joining method (Saitou and Nei, 1987) along with related type species were constructed using the software package MEGA version 5.2 (Tamura et al., 2011). Distances were calculated according to Kimura's two-parameter model (Kimura, 1983). To determine the support of each clade, bootstrap analysis was performed with 1000 resamplings (Felsenstein, 1985).

### Nucleotide accession numbers

The partial 16S rRNA gene sequences were deposited in GenBank with the following accession numbers: KP883248-KP883278.

### Antimicrobial screening

The indicator pathogens used for antimicrobial screening were: *Bacillus subtilis* MTCC 121, *Escherichia coli* MTCC 739, *Pseudomonas aeruginosa* DN1, *Candida albicans* MTCC 227, *Candida vaginitis* CV, *Curvularia oryzae* MTCC 2605, *Fusarium oxysporum* MTCC 287, *Helminthosporum oryzae* MTCC 3717, *Pyricularia oryzae* MTCC 1477, *Rhizoctonia oryzae-sativae* MTCC 2162 and *Rhizoctonia solani* MTCC 4633. All the test pathogens were procured from Microbial Type Culture Collection (MTCC), Institute of Microbial Technology (IMTECH), Chandigarh, India except for DN1 (lab collection) and CV (clinical isolate gifted from the Centre for DNA Fingerprinting and Diagnostics (CDFD), Hyderabad).

Antimicrobial assays against the bacterial and candidal strains were performed by agar well diffusion method (Hugo and Russell, 1983). Antifungal bioassay was done by dual culture technique (Khamna et al., 2009). The mycelial growth inhibition was calculated using the formula:

Percentage growth inhibition=[(C−T)/C]×100%

where, C = Radial growth of the test pathogen in the control plate, and T = Radial growth of the test pathogen in the test plate.

### Screening for biosynthetic genes

Three sets of degenerate primers were used for amplification of PKS-I, PKS-II and NRPS specific domains (Metsä-Ketalä et al., 1999; González et al., 2005). The primers used are listed in Table 1. PCR amplifications were performed in eppendorf mastercycler in a final volume of 25 μl containing 5 μl reaction buffer (with Mg^2+^) (10x) (Bioline, USA), 0.5 μl of each primer (100 μM) (IDT, USA), 2.0 μl of dNTPs mixture (2.5 mM) (Bioline, USA), 0.15 μl of *Taq* DNA polymerase (2.5 U/μl) (Bioline, USA), 2.5 μl DMSO (HiMedia, India), 11.85 μl deionized water and 2.5 μl of extracted DNA. Amplification was done using the following protocol: one denaturation step of 94°C for 5 min; 30 amplification cycles of 94°C for 1 min, 57°C (for K1F-M6R and A3F-A7R) or 58°C (for KSαF-KSαR) for 1 min, and 72°C for 2 min; and a final extension at 72°C for 5 min. Amplification products were analyzed in agarose gel (1%) using DNA ladder (100 bp) (Promega) as reference.

**Table 1:** **PCR primers for screening the biosynthetic genes**.

**Primer name**	**Sequence (5′–3′)**	**Target gene**	**Length of target gene fragment (bp)**	**References**
K1F M6R	TSA AGT CSA ACA TCG GBC A CGC AGG TTS CSG TAC CAG TA	PKS-I	1200–1400	González et al., 2005
KSαF KSαR	TSG CST GCT TGG AYG CSA TC TGG AAN CCG CCG AAB CCG CT	PKS-II	600	Metsä-Ketalä et al., 1999
A3F A7R	GCS TAC SYS ATS TAC ACS TCS GG SAS GTC VCC SGT SCG GTA S	NRPS	700–800	González et al., 2005

## Results

### Description of sampling sites

For the actinobacterial isolation, six Hundung samples were collected and used. The sample collection sites included: an abandoned cement factory site (Sample 1), quarry sites (Sample 2–5) and a rice field adjoining the quarry site (Sample 6). The limestones in Hundung, with color ranging from light gray to brown, are of good quality grade which are suitable for production of cement (Bhatt and Bhargava, 2005). The estimated reserve of this Hundung limestone is about 1.88 million tons (Sadangi, 2008; Lisam, 2011). The general characteristics of the samples used for isolation are highlighted in Table 2.

**Table 2:** **Profile of the Hundung limestone samples and coding scheme for the actinobacterial strains**.

**Sample**	**Sampling sites**	**pH of the sample**	**Isolation medium**	**No. of strains**	**Isolate code**
1	Cement factory location	9.26	GM1	29	MBRL 1–MBRL 29
			SCNA	22	MBRL 200–MBRL 221
2	Quarry site 1	8.70	GM1	33	MBRL 30–MBRL 61
			SCNA	15	MBRL 222–MBRL 237
3	Quarry site 2	7.45	GM1	–	–
			SCNA	10	MBRL 238–MBRL 247
4	Quarry site 3	6.50	GM1	14	MBRL 62–MBRL 75
			SCNA	3	MBRL 248–MBRL 250
5	Quarry site 4	7.91	GM1	–	–
			SCNA	2	MBRL 251–MBRL 252
6	Soil sample from the adjoining rice field	4.89	GM1	6	MBRL 76–MBRL 81
			SCNA	3	MBRL 253–MBRL 255
		Total number of strains		137	

### Actinobacterial isolation

Among the isolates obtained, 137 morphologically distinct putative actinobacterial strains were selected for further studies. These included 51 strains from Sample 1, 48 from Sample 2, 10 from Sample 3, 17 from Sample 4, 2 from Sample 5 and 9 from Sample 6. The coding scheme for the actinobacteria from the Hundung samples is shown in Table 2.

### Diversity analysis of Hundung actinobacteria

**Figure 1:**
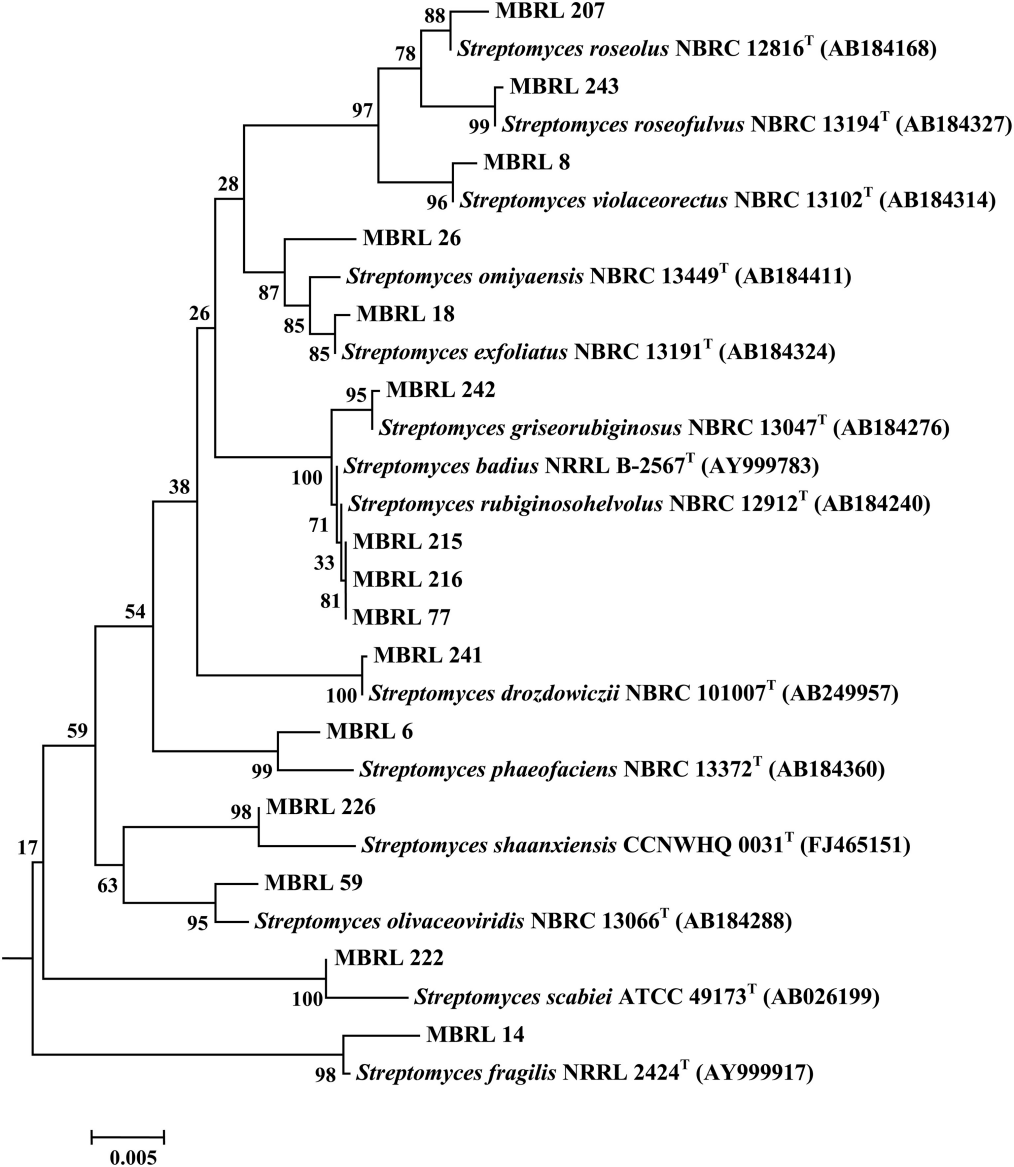
**Dendrogram of the representative *Streptomyces* strains based on the 16S rRNA gene sequences**. Numbers at nodes are levels of bootstrap support (%) for branch points (1000 resamplings). Bar, 0.002 substitutions per nucleotide position.

**Figure 2:**
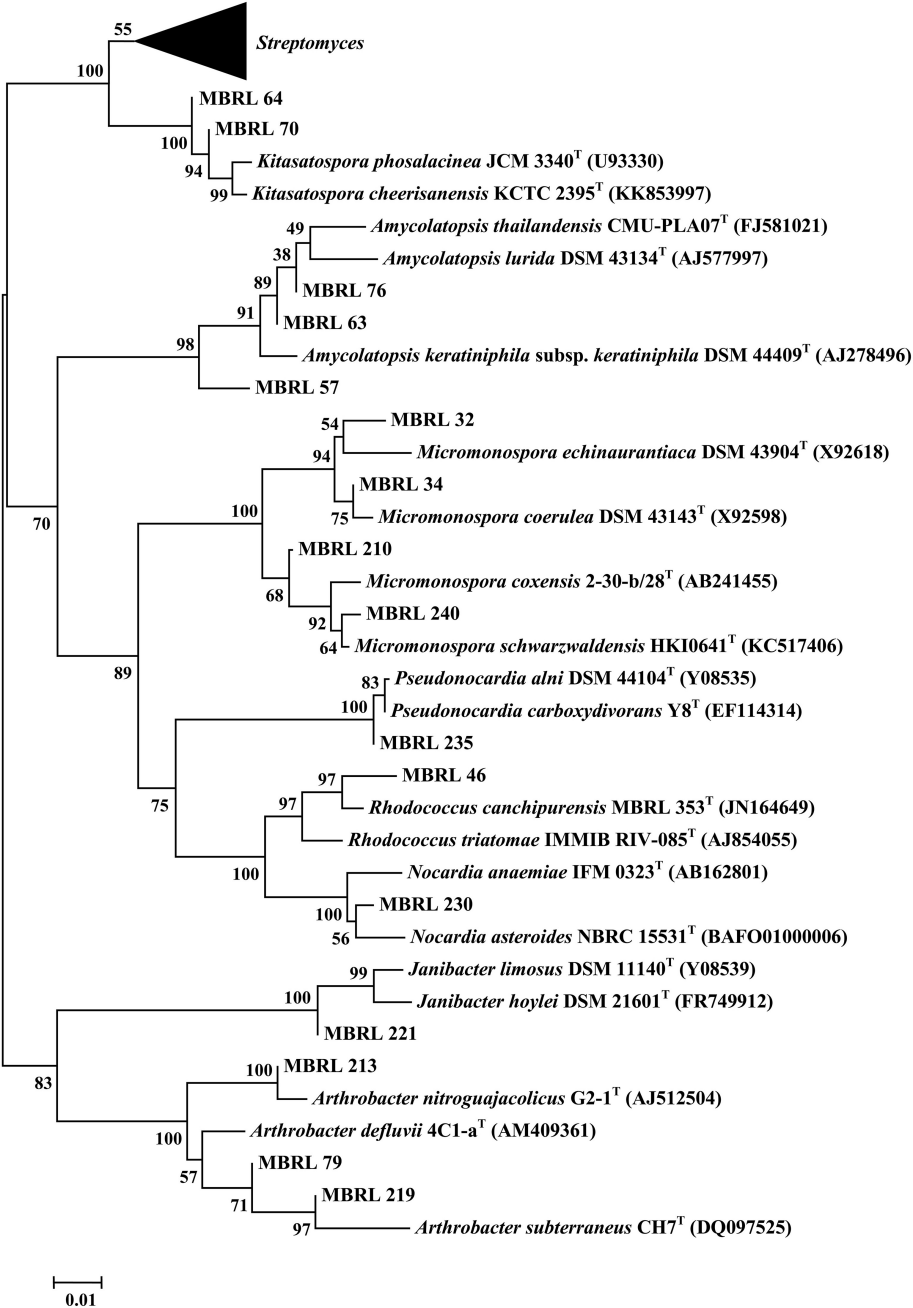
**Dendrogram of the representative rare actinobacterial strains based on the 16S rRNA gene sequences**. Numbers at nodes are levels of bootstrap support (%) for branch points (1000 resamplings). Bar, 0.01 substitutions per nucleotide position.

Upon analysis of the ARDRA-based dendrogram (Supplementary Figure S1), the isolates were classified into 31 phylotypic groups (see Supplementary Table S1 for classification pattern of the Hundung actinobacteria based on ARDRA-dendrogram). The 16S rRNA gene sequence profile for these 31 phylotypic groups is given in Table 3. Fifteen of these phylotypes belong to the genus *Streptomyces*. In addition, four phylotypes belong to the genus *Micromonospora*, three each to *Arthrobacter* and *Amycolatopsis* and two to *Kitasatospora*. Remaining phylotypes comprise of the genera *Janibacter, Rhodococcus, Nocardia*, and *Pseudonocardia*. Among the different sites, sample 2 gave the highest diversity (16 phylotypes), followed closely by sample 1 (15 phylotypic groups). Sample 1 yielded the genera *Streptomyces*, *Janibacter*, *Arthrobacter*, *Amycolatopsis*, and *Micromonospora* while sample 2 generated *Streptomyces*, *Rhodococcus*, *Amycolatopsis*, *Micromonospora*, *Arthrobacter*, *Nocardia*, and *Pseudonocardia*. Sample 3 which contained 5 phylogenetic groups yielded the genera *Streptomyces* and *Micromonospora*. Four genera viz., *Streptomyces*, *Janibacter*, *Amycolatopsis*, and *Kitasatospora* were present in sample 4 while sample 6 contained 3 genera: *Streptomyces*, *Amycolatopsis*, and *Arthrobacter*. Sample 5 yielded *Streptomyces* strains only though this may not reflect the true actinobacterial diversity in this sample, as we have selected only 2 strains from the isolates obtained from this sample. Nonetheless, overall analysis of the Hundung sites (1–6) indicated *Streptomyces* to be the dominant genus in these habitats. Figures 1, 2 depict the dendrograms based on the 16S rRNA gene sequences of the *Streptomyces* and rare actinobacterial strains obtained from Hundung limestone habitats.

**Table 3:** **Sequence analysis profile of representative strain of each phylotypic group**.

**Phylotypic group**	**Strain**	**Accession number**	**Closest homolog**	**Pairwise similarity (%)**
I	MBRL 216	KP883268	*Streptomyces badius* NRRL B-2567^T^	100.00
II	MBRL 221	KP883270	*Janibacter limosus* DSM 11140^T^	99.52
III	MBRL 6	KP883248	*Streptomyces phaeofaciens* NBRC 13372^T^	99.20
IV	MBRL 77	KP883262	*Streptomyces rubiginosohelvolus* NBRC 12912^T^	100.00
V	MBRL 207	KP883264	*Streptomyces roseolus* NBRC 12816^T^	99.74
VI	MBRL 241	KP883276	*Streptomyces drozdowiczii* NBRC 101007^T^	100.00
VII	MBRL 26	KP883252	*Streptomyces omiyaensis* NBRC 13449^T^	99.30
VIII	MBRL 243	KP883278	*Streptomyces roseofulvus* NBRC 13194^T^	100.00
IX	MBRL 213	KP883266	*Arthrobacter nitroguajacolicus* G2-1^T^	100.00
X	MBRL 219	KP883269	*Arthrobacter subterraneus* CH7^T^	98.96
XI	MBRL 46	KP883255	*Rhodococcus canchipurensis* MBRL 353^T^	98.45
XII	MBRL 57	KP883256	*Amycolatopsis lurida* DSM 43134^T^	97.46
XIII	MBRL 222	KP883271	*Streptomyces scabiei* ATCC 49173^T^	100.00
XIV	MBRL 76	KP883261	*Amycolatopsis thailandensis* CMU-PLA07^T^	98.95
XV	MBRL 32	KP883253	*Micromonospora kangleipakensis* MBRL 34^T^	98.42
XVI	MBRL 64	KP883259	*Kitasatospora phosalacinea* JCM 3340^T^	99.35
XVII	MBRL 70	KP883260	*Kitasatospora cheerisanensis* KCTC 2395^T^	99.40
XVIII	MBRL 210	KP883265	*Micromonospora coxensis* 2-30-b/28^T^	99.27
XIX	MBRL 240	KP883275	*Micromonospora schwarzwaldensis* HKI0641^T^	99.48
XX	MBRL 8	KP883249	*Streptomyces violacerectus* NBRC 13102^T^	100.00
XXI	MBRL 18	KP883251	*Streptomyces exfoliatus* NBRC 13191^T^	100.00
XXII	MBRL 14	KP883250	*Streptomyces fragilis* NRRL 2424^T^	99.44
XXIII	MBRL 63	KP883258	*Amycolatopsis keratiniphila* subsp. *keratiniphila* DSM 44409^T^	99.92
XXIV	MBRL 34	KP883254	*Micromonospora coerulea* DSM 43143^T^	98.52
XXV	MBRL 242	KP883277	*Streptomyces griseorubiginosus* NBRC 13047^T^	100.00
XXVI	MBRL 226	KP883272	*Streptomyces shaanxiensis* CCNWHQ 0031^T^	99.52
XXVII	MBRL 215	KP883267	*Streptomyces rubiginosohelvolus* NBRC 12912^T^	100.00
XXVIII	MBRL 79	KP883263	*Arthrobacter defluvii* 4C1-a^T^	100.00
XXIX	MBRL 230	KP883273	*Nocardia asteroides* NBRC 15531^T^	98.64
XXX	MBRL 235	KP883274	*Pseudonocardia carboxydivorans* Y8^T^	100.00
XXXI	MBRL 59	KP883257	*Streptomyces olivaceoviridis* NBRC 13066^T^	99.49

### Antimicrobial activities

#### Antibacterial and anticandidal activities

Antibacterial and anticandidal activity was assessed against a set of indicator organisms. The antibacterial and anticandidal profiles of the Hundung actinobacteria are shown in Table 4. Of 137 actinobacterial isolates, 19 exhibited antimicrobial activities against at least one of the test pathogens. In case of *Bacillus subtilis*, 18 strains showed inhibition, of which 5 (MBRL 5, MBRL 10, MBRL 201, MBRL 204, MBRL 251) showed inhibition zones above 17 mm diameter. Against *Escherichia coli*, 5 strains exhibited antagonistic activities of which 2 (MBRL 5, MBRL 10) showed inhibition zone sizes above 17 mm diameter. No strain had activity against *Pseudomonas aeruginosa*. Against *Candida albicans*, 5 strains showed inhibitory activity and 4 against *Candida vaginitis* (See Supplementary Table S2 for complete antibacterial and anticandidal profile).

**Table 4:** **Antimicrobial, biocontrol and biosynthetic genes profile of the Hundung actinobacteria**.

**Phylotypic group**	**Total number of isolates**	**Genus classified**	**Test pathogens**											**Biosynthetic gene**		
			**MTCC 121**	**MTCC 739**	**DN1**	**MTCC 227**	**CV**	**MTCC 2605**	**MTCC 287**	**MTCC 3717**	**MTCC 1477**	**MTCC 2162**	**MTCC 4633**	**PKS-I**	**PKS-II**	**NRPS**
I	29	*Streptomyces*	4	4	0	4	3	5	7	12	8	10	10	4	24	23
II	2	*Janibacter*	1	0	0	0	0	1	0	1	1	1	1	0	1	1
III	27	*Streptomyces*	4	0	0	1	1	3	2	4	5	5	6	14	24	19
IV	1	*Streptomyces*	1	0	0	0	0	0	0	1	1	0	0	0	1	1
V	2	*Streptomyces*	0	0	0	0	0	0	0	0	0	0	0	2	2	2
VI	4	*Streptomyces*	0	0	0	0	0	0	0	1	0	0	0	1	3	2
VII	1	*Streptomyces*	0	0	0	0	0	0	0	1	0	0	0	1	1	1
VIII	1	*Streptomyces*	0	0	0	0	0	0	0	0	0	0	0	1	2	0
IX	1	*Arthrobacter*	0	0	0	0	0	0	0	0	0	0	0	1	1	1
X	1	*Arthrobacter*	0	0	0	0	0	0	0	0	0	0	0	0	0	0
XI	1	*Rhdococcus*	0	0	0	0	0	1	0	0	0	0	0	0	0	0
XII	17	*Amycolatopsis*	6	0	0	0	0	2	2	4	3	5	5	9	9	16
XIII	1	*Streptomyces*	0	0	0	0	0	0	0	0	0	0	0	0	0	1
XIV	1	*Amycolatopsis*	0	0	0	0	0	0	0	0	0	0	0	0	0	1
XV	2	*Micromonospora*	0	0	0	0	0	0	0	0	0	0	0	0	1	0
XVI	1	*Kitasatospora*	0	0	0	0	0	0	0	0	0	0	0	0	1	0
XVII	2	*Kitasatospora*	1	0	0	0	0	0	0	0	0	0	0	0	2	2
XVIII	1	*Micromonospora*	0	0	0	0	0	0	0	0	0	0	0	0	0	0
XIX	1	*Micromonospora*	0	0	0	0	0	0	0	0	0	0	0	2	0	0
XX	1	*Streptomyces*	0	0	0	0	0	1	1	1	0	0	0	1	0	0
XXI	2	*Streptomyces*	0	0	0	0	0	1	0	1	0	0	1	1	1	1
XXII	7	*Streptomyces*	0	0	0	0	0	0	0	2	0	1	1	3	4	1
XXIII	1	*Amycolatopsis*	0	0	0	0	0	1	0	1	1	1	1	1	1	1
XXIV	1	*Micromonospora*	0	0	0	0	0	0	0	0	0	0	0	0	1	0
XXV	18	*Streptomyces*	1	1	0	0	0	0	1	2	0	0	0	3	11	3
XXVI	1	*Streptomyces*	0	0	0	0	0	0	0	0	0	0	0	0	1	0
XXVII	2	*Streptomyces*	0	0	0	0	0	0	0	0	0	0	0	1	1	1
XXVIII	2	*Arthrobacter*	0	0	0	0	0	0	0	0	0	0	0	0	3	1
XXIX	1	*Nocardia*	0	0	0	0	0	0	0	0	0	0	0	0	0	0
XXX	2	*Pseudonocardia*	0	0	0	0	0	0	0	0	1	0	0	0	4	0
XXXI	3	*Streptomyces*	0	0	0	0	0	0	0	0	0	0	0	2	2	2

*MTCC 121, Bacillus subtilis; MTCC 739, Escherichia coli; DN1, Pseudomonas aeruginosa; MTCC 227, Candida albicans; CV, Candida vaginitis; MTCC 2605, Curvularia oryzae; MTCC 287, Fusarium oxysporum; MTCC 3717, Helminthosporum oryzae; MTCC 1477, Pyricularia oryzae; MTCC 2162, Rhizoctonia oryzae-sativae; MTCC 4633, Rhizoctonia solani*.

#### Biocontrol activities

Several actinobacterial strains exhibited biocontrol potential against rice fungal pathogens. Forty five actinobacterial strains from Hundung limestone habitat showed biocontrol activities against at least one of the rice fungal pathogens. Frequencies of biocontrol activities against the indicator fungal pathogens were as follows: *Helminthosporum oryzae* MTCC 3717 (22.6%), *Rhizoctonia solani* MTCC 4633 (18.2%), *Rhizoctonia oryzae-sativae* MTCC 2162 (16.8%), *Pyricularia oryzae* MTCC 1477 (14.6%), *Curvularia oryzae* MTCC 2605 (10.9%) and *Fusarium oxysporum* MTCC 287 (9.5%) respectively. Table 4 summarizes the biocontrol profile of the Hundung actinobacteria (See Supplementary Table S3 for complete biocontrol activity profile).

### Screening for biosynthetic genes

It is well known that many bioactive metabolites in actinobacteria are produced by PKS and NRPS gene clusters. Screening for genes associated with secondary metabolism is helpful in evaluating the biosynthetic potential of actinobacteria. Of 137 Hundung actinobacterial strains, 118 possessed at least one of the three biosynthetic gene clusters. A total of 43 strains had a single type of biosynthetic gene cluster (PKS-I, 5 strains; PKS-II, 27; NRPS, 11). The remaining 75 strains had two or more of the biosynthetic gene clusters: 9 strains possessed both PKS-I and PKS-II; 8 had both PKS-I and NRPS; 36 had both PKS-II and NRPS while 22 strains had all the three biosynthetic gene clusters. Table 4 shows the amplication profile for biosynthetic genes in the Hundung actinobacteria (see Supplementary Table S4 for complete PCR profile of biosynthetic genes).

## Discussion

Diversity profiling focused on actinobacteria in limestone habitats started when Kim et al. (1998) reported the diversity of actinobacteria antagonistic to phytopathogenic fungi in caves of Korea. They reported the presence of *Streptomyces, Micromonospora*, Nocardioform actinobacteria, *Actinomyces*, *Dactylosporangium*, *Saccharomonospora*, and *Streptosporangium* in these habitats. Groth et al. (1999a) studied the actinobacterial diversity in Karstic caves (Altamira and Tito Bustillo) located in northern Spain and reported members of the genera *Streptomyces*, *Nocardia*, *Rhodococcus*, *Nocardioides*, *Amycolatopsis*, *Saccharothrix*, *Brevibacterium*, *Microbacterium*, and coccocid actinobacteria of the family *Micrococcaceae*. Groth et al. (2002) reported the isolation of a new genus *Knoellia* from limestone caves. To the repertoire of the actinobacterial diversity in caves, Nakaew et al. (2009) added the genera *Nonomuraea*, *Actinocorallia*, *Catellatospora*, *Microbispora*, and *Sprillospora*. Jurado et al. (2009) reported the new genus *Hoyosella* from cave biofilms in Spain. Niyomvong et al. (2012) found the presence of the genera *Streptomyces*, *Actinomadura*, *Actinoplanes*, *Gordonia*, *Microbispora*, *Micromonospora*, *Nocardia*, *Nonomuraea*, and *Saccharopolyspora* in the tropical limestone caves of Khao No-Khao Kaeo karst in Thailand.

Considering the rich diversity of actinobacteria in limestone habitats, the present study on actinobacterial diversity of limestone deposit sites in Hundung, Manipur, India, has special significance. As per our findings, the genus *Streptomyces* is predominantly present in these limestone habitats. This is also indicated by the presence of phylotypic group III (represented by the genus *Streptomyces*) in all the six samples used for actinobacterial isolation. Apart from *Streptomyces*, we also observed the presence of rare actinobacteria *Micromonospora*, *Arthrobacter*, *Amycolatopsis*, *Kitasatospora*, *Janibacter*, *Rhodococcus*, *Nocardia*, and *Pseudonocardia*. This work forms the first report of the isolation of *Janibacter* and *Kitasatospora* from limestone and related habitats.

ARDRA is preferable to other molecular genome typing methods for preliminary phylogenetic grouping as it is faster and more cost effective than the other approaches. Moreover, as ARDRA is based on the presence of restriction sites within the ribosomal DNA, duplicate strains will most likely have the same restriction pattern. The use of ARDRA in this study, therefore, helped reduce the number of duplicate strains among the isolates from the community, indicating the true diversity of the community even when the sample size is small.

In the course of a screening program for novel antibiotics from strains obtained from Grotta dei Cervi, a cave in Italy, Herold et al. (2004) identified a bioactive complex, Cervimycins A–D, from a strain of *Streptomyces tendae*. Cervimycins are potent antibiotics against multidrug resistant *Staphylococcus aureus* (MRSA) and vancomycin-resistant *Enterococcus faecalis* (VRE) strains (Herold et al., 2005). Quadri and Agsar (2012) have investigated antimicrobial activities of actinobacteria of limestone quarries located at Deccan traps, India. Of 63 actinobacteria from this habitat, six strains (belonging to the genera *Streptomyces*, *Micromonospora*, *Nonomuraea*, *Kribbella*, *Lechevalieria*, and *Saccharothrix*) showed potent antimicrobial activity against *Bacillus subtilis*, *Escherichia coli*, *Klebsiella pneumoniae*, *Pseudomonas aeruginosa*, *Staphylococcus aureus*, *Salmonella typhi*, and *Candida albicans*. Carlsohn (2011) found novel strains of *Amycolatopsis saalfeldensis*, *Kribbella aluminosa*, and *Streptomyces* strains from a mine in Germany and they were strongly inhibitory to *Stapthylococcus aureus*, *Mycobacterium smegmatis*, and *Candida albicans*, and moderately antagonistic to *Escherichia coli*. Rule and Cheeptham (2013) reported some *Streptomyces* strains from a volcanic cave in Canada (Cheeptham et al., 2013) as antagonistic to *Micrococcus luteus*, MRSA, *Mycobacterium smegmatis*, *Pseudomonas aeruginosa*, *Escherichia coli* and *Candida albicans*.

In the present study, 5 Hundung actinobacteria were found to be potent antimicrobial strains. Of these, 2 *Streptomyces* species MBRL 201 and MBRL 251 showed strong antimicrobial activity against *Bacillus subtilis*, but less bioactivity against *Escherichia coli, Candida albicans*, and *Candida vaginitis*. Besides these two, two other Hundung *Streptomyces* species (MBRL 5 and MBRL 10) also exhibited promising antimicrobial activities. MBRL 204, another Hundung *Streptomyces* strain, exhibited relatively lesser antimicrobial activity compared to the other 4 isolates (MBRL 5, MBRL 10, MBRL 201 and MBRL 251).

Soil actinobacteria have been proposed as promising biocontrol agents (Goodfellow and Williams, 1983; Chater, 1993). However, actinobacteria from limestone habitats have not been investigated for their biocontrol potential. Quadri and Agsar (2012) have reported that only 9.5% of the strains isolated from limestone habitats have antifungal activities against *Aspergillus fumigates*, *Aspergillus niger*, and *Fusarium solani*. In the current investigation, many strains belonging to the genera *Streptomyces* and *Amycolatopsis* (e.g., Phylotypic group I, III and XII) were found to have biocontrol activities against selected rice fungal pathogens *Curvularia oryzae*, *Fusarium oxysporum*, *Helminsthosporum oryzae*, *Pyricularia oryzae*, *Rhizoctonia oryzae-sativae*, and *Rhizoctonia solani*. Rare actinobacteria belonging to genera *Janibacter* and *Pseudonocardia* obtained from Hundung limestone habitats also exhibited significant biocontrol potential against some fungal pathogens.

The biosynthetic gene clusters play a crucial role in microbial natural product biosynthesis. The biosynthesis of cervimycin complex (metabolites reported from limestone related habitats) involved the type II PKS system. Other antibacterial metabolites such as Ravidomycins from *Streptomyces rabidus* are biosynthesized by type II PKS system (Kharel et al., 2010). Hence, it is imperative to screen for the presence of these biosynthetic gene clusters in the actinobacterial isolates. In our studies, 118 of the 137 actinobacterial isolates were found to have at least one of the three biosynthetic gene clusters. Of these 118 actinobacteria possessed the biosynthetic gene clusters, 47 exhibited antimicrobial and/or biocontrol activities indicating that less than 50% of the strains possessing biosynthetic gene clusters were bioactive under the screening condition. The findings of the various experiments indicate that 86% of the strains isolated from Hundung limestone rocks possessed biosynthetic gene clusters of which 40% exhibited antimicrobial activities. It can, therefore, be concluded that limestone habitats is a promising source for search of novel secondary metabolites.

## Author contributions

SN planned, conducted the experiments, analyzed the data, and prepared the manuscript, AD performed, analyzed and interpreted the ARDRA data, KT performed the biocontrol assay, DN and WL supervised the experiments.

### Conflict of interest statement

The authors declare that the research was conducted in the absence of any commercial or financial relationships that could be construed as a potential conflict of interest.

## Abbreviations

ARDRA: Amplified Ribosomal DNA Restriction Analysis
DGGE: Denaturing Gradient Gel Electrophoresis
GM1: Gauze Medium No. 1
NRPS: Non-Ribosomal Peptide Synthetase
PKS: Polyketide Synthase
RFLP: Restriction Fragment Length Polymorphism
SCNA: Starch Casein Nitrate Agar.
