# Supplementary material for: Actinobacterial diversity in limestone deposit sites in Hundung, Manipur (India) and their antimicrobial activities
Source: Front Microbiol. 2015 May 5;6:413. doi: 10.3389/fmicb.2015.00413 (PMC4419841; doi:10.3389/fmicb.2015.00413)
Supplement: Supplementary file 1 [file Table1.DOCX]

***Supplementary Material***

**Actinobacterial diversity in limestone deposit sites in Hundung, Manipur (India) and their antimicrobial activities**

**Salam Nimaichand^1,2^*, Asem Mipeshwaree Devi^3^, K. Tamreihao^1^, Debananda S. Ningthoujam^1^, Wen-Jun Li^2,4^***

^1^Microbial Biotechnology Research Laboratory, Department of Biochemistry, Manipur University, Canchipur, Imphal, Manipur, India

^2^State Key Laboratory of Biocontrol and Guangdong Key Laboratory of Plant Resources, School of Life Sciences, Sun Yat-Sen University, Guangzhou, China

^3^Molecular Genetics Laboratory, Department of Botany, North-Eastern Hill University, Shillong, Meghalaya, India

^4^Yunnan Institute of Microbiology, Yunnan University, Kunming, China

***Correspondence: Salam Nimaichand,** Department of Biochemistry, Manipur University,Canchipur, Imphal – 795003Manipur, India

Email: [s.nimaichand@gmail.com](mailto:s.nimaichand@gmail.com)

**Wen-Jun Li**

Email: liwenjun3@mail.sysu.edu.cn

**Supplementary Table S1** Classification pattern of the Hundung actinobacterial strains based on ARDRA-dendrogram

| **Phylotypic group** | **Isolation medium** | **Strains** | **No. of strains** | **Total no. of strains** |
| --- | --- | --- | --- | --- |
| I | GM1 | MBRL 1, MBRL 2, MBRL 3, MBRL 5, MBRL 7, MBRL 9,  MBRL 10, MBRL 11, MBRL 12, MBRL 19, , MBRL 21, MBRL 23, MBRL 27, MBRL 28, MBRL 29, MBRL 31, MBRL 39, MBRL 43, MBRL 44, MBRL 47, MBRL 48, MBRL 49, MBRL 50, MBRL 55, MBRL 56, MBRL 60, MBRL 71 | 27 | 29 |
|  | SCNA | MBRL 201, MBRL 216 | 2 |  |
| II | GM1 | MBRL 67 | 1 | 2 |
|  | SCNA | MBRL 221 | 1 |  |
| III | GM1 | MBRL 4, MBRL 6, MBRL 16, MBRL 22, MBRL 24, MBRL 30, MBRL 33, MBRL 37, MBRL 41, MBRL 45, MBRL 51, MBRL 54, MBRL 73, MBRL 74, MBRL 78, MBRL 80, MBRL 81 | 17 | 27 |
|  | SCNA | MBRL 200, MBRL 202, MBRL 203, MBRL 214,  MBRL 225, MBRL 238, MBRL 248, MBRL 250,  MBRL 251, MBRL 252 | 10 |  |
| IV | GM1 | MBRL 77 | 1 | 1 |
|  | SCNA | - | 0 |  |
| V | GM1 | - | 0 | 2 |
|  | SCNA | MBRL 206, MBRL 207 | 2 |  |
| VI | GM1 | - | 0 | 4 |
|  | SCNA | MBRL 227, MBRL 228, MBRL 241, MBRL 245 | 4 |  |
| VII | GM1 | MBRL 26 | 1 | 1 |
|  | SCNA | - | 0 |  |
| VIII | GM1 | - | 0 | 1 |
|  | SCNA | MBRL 243 | 1 |  |
| IX | GM1 | - | 0 | 1 |
|  | SCNA | MBRL 213 | 1 |  |
| X | GM1 | - | 0 | 1 |
|  | SCNA | MBRL 219 | 1 |  |
| XI | GM1 | MBRL 46 | 1 | 1 |
|  | SCNA | - | 0 |  |
| XII | GM1 | MBRL 13, MBRL 17, MBRL 20, MBRL 35, MBRL 36, MBRL 38, MBRL 40, MBRL 53, MBRL 57, MBRL 58, MBRL 62, MBRL 65, MBRL 66, MBRL 68, MBRL 69, MBRL 72 | 16 | 17 |
|  | SCNA | MBRL 234 | 1 |  |
| XIII | GM1 | - | 0 | 1 |
|  | SCNA | MBRL 222 | 1 |  |
| XIV | GM1 | MBRL 76 | 1 | 1 |
|  | SCNA | - | 0 |  |
| XV | GM1 | MBRL 32 | 1 | 2 |
|  | SCNA | MBRL 211 | 1 |  |
| XVI | GM1 | MBRL 64 | 1 | 1 |
|  | SCNA | - | 0 |  |
| XVII | GM1 | MBRL 70, MBRL 75 | 2 | 2 |
|  | SCNA | - | 0 |  |
| XVIII | GM1 | - | 0 | 1 |
|  | SCNA | MBRL 210 | 1 |  |
| XIX | GM1 | - | 0 | 1 |
|  | SCNA | MBRL 240 | 1 |  |
| XX | GM1 | MBRL 8 | 1 | 1 |
|  | SCNA | - | 0 |  |
| XXI | GM1 | MBRL 18, | 1 | 2 |
|  | SCNA | MBRL 232 | 1 |  |
| XXII | GM1 | MBRL 14, MBRL 15, MBRL 25, MBRL 61 | 4 | 7 |
|  | SCNA | MBRL 253, MBRL 254, MBRL 255 | 3 |  |
| XXIII | GM1 | MBRL 63 | 1 | 1 |
|  | SCNA | - | 0 |  |
| XXIV | GM1 | MBRL 34 | 1 | 1 |
|  | SCNA | - | 0 |  |
| XXV | GM1 | - | 0 | 18 |
|  | SCNA | MBRL 204, MBRL 205, MBRL 208, MBRL 209,  MBRL 212, MBRL 217, MBRL 218, MBRL 223,  MBRL 224, MBRL 229, MBRL 231, MBRL 233,  MBRL 239, MBRL 242, MBRL 244, MBRL 246,  MBRL 247, MBRL 249 | 18 |  |
| XXVI | GM1 | - | 0 | 1 |
|  | SCNA | MBRL 226 | 1 |  |
| XXVII | GM1 | - | 0 | 2 |
|  | SCNA | MBRL 215, MBRL 220 | 2 |  |
| XXVIII | GM1 | MBRL 79 | 1 | 2 |
|  | SCNA | MBRL 237 | 1 |  |
| XXIX | GM1 | - | 0 | 1 |
|  | SCNA | MBRL 230 | 1 |  |
| XXX | GM1 | MBRL 235, MBRL 236 | 2 | 2 |
|  | SCNA | - | 0 |  |
| XXXI | GM1 | MBRL 42, MBRL 52, MBRL 59 | 3 | 3 |
|  | SCNA | - | 0 |  |
|  |  | Total isolates |  | 137 |
