# Supplementary material for: Actinobacterial diversity in limestone deposit sites in Hundung, Manipur (India) and their antimicrobial activities
Source: Front Microbiol. 2015 May 5;6:413. doi: 10.3389/fmicb.2015.00413 (PMC4419841; doi:10.3389/fmicb.2015.00413)
Supplement: Supplementary file 2 [file Table2.DOCX]

***Supplementary Material***

**Actinobacterial diversity in limestone deposit sites in Hundung, Manipur (India) and their antimicrobial activities**

**Salam Nimaichand^1,2^*, Asem Mipeshwaree Devi^3^, K. Tamreihao^1^, Debananda S. Ningthoujam^1^, Wen-Jun Li^2,4^***

^1^Microbial Biotechnology Research Laboratory, Department of Biochemistry, Manipur University, Canchipur, Imphal, Manipur, India

^2^State Key Laboratory of Biocontrol and Guangdong Key Laboratory of Plant Resources, School of Life Sciences, Sun Yat-Sen University, Guangzhou, China

^3^Molecular Genetics Laboratory, Department of Botany, North-Eastern Hill University, Shillong, Meghalaya, India

^4^Yunnan Institute of Microbiology, Yunnan University, Kunming, China

***Correspondence: Salam Nimaichand,** Department of Biochemistry, Manipur University,Canchipur, Imphal – 795003Manipur, India

Email: [s.nimaichand@gmail.com](mailto:s.nimaichand@gmail.com)

**Wen-Jun Li**

Email: liwenjun3@mail.sysu.edu.cn

**Supplementary Table S2** Antimicrobial profile of Hundung actinobacterial strains against bacterial and candidal pathogens

| ***Strain*** | ***Inhibition zone*** | | | | | ***Phylotypic type*** |
| --- | --- | --- | --- | --- | --- | --- |
|  | ***MTCC 121*** | ***MTCC 739*** | ***DN1*** | ***MTCC 227*** | ***CV*** |  |
| MBRL 1 | - | - | - | - | - | I |
| MBRL 2 | - | - | - | - | - | I |
| MBRL 3 | - | - | - | - | - | I |
| MBRL 4 | - | - | - | - | - | III |
| MBRL 5 | ++ | ++ | - | + | + | I |
| MBRL 6 | - | - | - | - | - | III |
| MBRL 7 | - | - | - | - | - | I |
| MBRL 8 | - | - | - | - | - | XX |
| MBRL 9 | - | - | - | - | - | I |
| MBRL 10 | ++ | ++ | - | + | + | I |
| MBRL 11 | - | - | - | - | - | I |
| MBRL 12 | - | - | - | - | - | I |
| MBRL 13 | - | - | - | - | - | XII |
| MBRL 14 | - | - | - | - | - | XXII |
| MBRL 15 | - | - | - | - | - | XXII |
| MBRL 16 | - | - | - | - | - | III |
| MBRL 17 | - | - | - | - | - | XII |
| MBRL 18 | - | - | - | - | - | XXI |
| MBRL 19 | - | - | - | - | - | I |
| MBRL 20 | + | - | - | - | - | XII |
| MBRL 21 | - | - | - | - | - | I |
| MBRL 22 | - | - | - | - | - | III |
| MBRL 23 | - | - | - | - | - | I |
| MBRL 24 | - | - | - | - | - | III |
| MBRL 25 | - | - | - | - | - | XXII |
| MBRL 26 | - | - | - | - | - | VII |
| MBRL 27 | + | + | - | - | - | I |
| MBRL 28 | - | - | - | + | - | I |
| MBRL 29 | - | - | - | - | - | I |
| MBRL 30 | - | - | - | - | - | III |
| MBRL 31 | - | - | - | - | - | I |
| MBRL 32 | - | - | - | - | - | XV |
| MBRL 33 | - | - | - | - | - | III |
| MBRL 34 | - | - | - | - | - | XXIV |
| MBRL 35 | - | - | - | - | - | XII |
| MBRL 36 | - | - | - | - | - | XII |
| MBRL 37 | - | - | - | - | - | III |
| MBRL 38 | - | - | - | - | - | XII |
| MBRL 39 | - | - | - | - | - | I |
| MBRL 40 | + | - | - | - | - | XII |
| MBRL 41 | - | - | - | - | - | III |
| MBRL 42 | - | - | - | - | - | XXXI |
| MBRL 43 | - | - | - | - | - | I |
| MBRL 44 | - | - | - | - | - | I |
| MBRL 45 | - | - | - | - | - | III |
| MBRL 46 | - | - | - | - | - | XI |
| MBRL 47 | - | - | - | - | - | I |
| MBRL 48 | - | - | - | - | - | I |
| MBRL 49 | - | - | - | - | - | I |
| MBRL 50 | - | - | - | - | - | I |
| MBRL 51 | - | - | - | - | - | III |
| MBRL 52 | - | - | - | - | - | XXXI |
| MBRL 53 | - | - | - | - | - | XII |
| MBRL 54 | - | - | - | - | - | III |
| MBRL 55 | - | - | - | - | - | I |
| MBRL 56 | - | - | - | - | - | I |
| MBRL 57 | + | - | - | - | - | XII |
| MBRL 58 | - | - | - | - | - | XII |
| MBRL 59 | - | - | - | - | - | XXXI |
| MBRL 60 | - | - | - | - | - | I |
| MBRL 61 | - | - | - | - | - | XXII |
| MBRL 62 | - | - | - | - | - | XII |
| MBRL 63 | - | - | - | - | - | XXIII |
| MBRL 64 | - | - | - | - | - | XVI |
| MBRL 65 | - | - | - | - | - | XII |
| MBRL 66 | + | - | - | - | - | XII |
| MBRL 67 | + | - | - | - | - | II |
| MBRL 68 | + | - | - | - | - | XII |
| MBRL 69 | + | - | - | - | - | XII |
| MBRL 70 | + | - | - | - | - | XVII |
| MBRL 71 | - | - | - | - | - | I |
| MBRL 72 | - | - | - | - | - | XII |
| MBRL 73 | - | - | - | - | - | III |
| MBRL 74 | + | - | - | - | - | III |
| MBRL 75 | - | - | - | - | - | XVII |
| MBRL 76 | - | - | - | - | - | XIV |
| MBRL 77 | + | - | - | - | - | IV |
| MBRL 78 | + | - | - | - | - | III |
| MBRL 79 | - | - | - | - | - | XXVIII |
| MBRL 80 | - | - | - | - | - | III |
| MBRL 81 | - | - | - | - | - | III |
| MBRL 200 | + | - | - | - | - | III |
| MBRL 201 | ++ | + | - | + | + | I |
| MBRL 202 | - | - | - | - | - | III |
| MBRL 203 | - | - | - | - | - | III |
| MBRL 204 | ++ | + | - | - | - | XXV |
| MBRL 205 | - | - | - | - | - | XXV |
| MBRL 206 | - | - | - | - | - | V |
| MBRL 207 | - | - | - | - | - | V |
| MBRL 208 | - | - | - | - | - | XXV |
| MBRL 209 | - | - | - | - | - | XXV |
| MBRL 210 | - | - | - | - | - | XVIII |
| MBRL 211 | - | - | - | - | - | XV |
| MBRL 212 | - | - | - | - | - | XXV |
| MBRL 213 | - | - | - | - | - | IX |
| MBRL 214 | - | - | - | - | - | III |
| MBRL 215 | - | - | - | - | - | XXVII |
| MBRL 216 | - | - | - | - | - | I |
| MBRL 217 | - | - | - | - | - | XXV |
| MBRL 218 | - | - | - | - | - | XXV |
| MBRL 219 | - | - | - | - | - | X |
| MBRL 220 | - | - | - | - | - | XXVII |
| MBRL 221 | - | - | - | - | - | II |
| MBRL 222 | - | - | - | - | - | XIII |
| MBRL 223 | - | - | - | - | - | XXV |
| MBRL 224 | - | - | - | - | - | XXV |
| MBRL 225 | - | - | - | - | - | III |
| MBRL 226 | - | - | - | - | - | XXVI |
| MBRL 227 | - | - | - | - | - | VI |
| MBRL 228 | - | - | - | - | - | VI |
| MBRL 229 | - | - | - | - | - | XXV |
| MBRL 230 | - | - | - | - | - | XXIX |
| MBRL 231 | - | - | - | - | - | XXV |
| MBRL 232 | - | - | - | - | - | XXI |
| MBRL 233 | - | - | - | - | - | XXV |
| MBRL 234 | - | - | - | - | - | XII |
| MBRL 235 | - | - | - | - | - | XXX |
| MBRL 236 | - | - | - | - | - | XXX |
| MBRL 237 | - | - | - | - | - | XXVIII |
| MBRL 238 | - | - | - | - | - | III |
| MBRL 239 | - | - | - | - | - | XXV |
| MBRL 240 | - | - | - | - | - | XIX |
| MBRL 241 | - | - | - | - | - | VI |
| MBRL 242 | - | - | - | - | - | XXV |
| MBRL 243 | - | - | - | - | - | VIII |
| MBRL 244 | - | - | - | - | - | XXV |
| MBRL 245 | - | - | - | - | - | VI |
| MBRL 246 | - | - | - | - | - | XXV |
| MBRL 247 | - | - | - | - | - | XXV |
| MBRL 248 | - | - | - | - | - | III |
| MBRL 249 | - | - | - | - | - | XXV |
| MBRL 250 | - | - | - | - | - | III |
| MBRL 251 | ++ | - | - | + | + | III |
| MBRL 252 | - | - | - | - | - | III |
| MBRL 253 | - | - | - | - | - | XXII |
| MBRL 254 | - | - | - | - | - | XXII |
| MBRL 255 | - | - | - | - | - | XXII |

Note: ++ 🡪 Above 17 mm diameter; + 🡪 Below 17 mm diameter
