# Supplementary material for: Actinobacterial diversity in limestone deposit sites in Hundung, Manipur (India) and their antimicrobial activities
Source: Front Microbiol. 2015 May 5;6:413. doi: 10.3389/fmicb.2015.00413 (PMC4419841; doi:10.3389/fmicb.2015.00413)
Supplement: Supplementary file 4 [file Table4.DOCX]

***Supplementary Material***

**Actinobacterial diversity in limestone deposit sites in Hundung, Manipur (India) and their antimicrobial activities**

**Salam Nimaichand^1,2^*, Asem Mipeshwaree Devi^3^, K. Tamreihao^1^, Debananda S. Ningthoujam^1^, Wen-Jun Li^2,4^***

^1^Microbial Biotechnology Research Laboratory, Department of Biochemistry, Manipur University, Canchipur, Imphal, Manipur, India

^2^State Key Laboratory of Biocontrol and Guangdong Key Laboratory of Plant Resources, School of Life Sciences, Sun Yat-Sen University, Guangzhou, China

^3^Molecular Genetics Laboratory, Department of Botany, North-Eastern Hill University, Shillong, Meghalaya, India

^4^Yunnan Institute of Microbiology, Yunnan University, Kunming, China

***Correspondence: Salam Nimaichand,** Department of Biochemistry, Manipur University, Canchipur, Imphal – 795003Manipur, India

Email: [s.nimaichand@gmail.com](mailto:s.nimaichand@gmail.com)

**Wen-Jun Li**

Email: liwenjun3@mail.sysu.edu.cn

**Supplementary Table S4** PCR profile of the biosynthetic genes for the Hundung actinobacterial strains

| ***Strain*** |  | ***PKS I*** | ***PKS II*** | ***NRPS*** |  | ***Phylotypic type*** |
| --- | --- | --- | --- | --- | --- | --- |
|  |  |  |  |  |  |  |
| MBRL 1 |  | - | - | - |  | I |
| MBRL 2 |  | - | + | + |  | I |
| MBRL 3 |  | - | + | + |  | I |
| MBRL 4 |  | - | + | + |  | III |
| MBRL 5 |  | - | + | + |  | I |
| MBRL 6 |  | - | + | + |  | III |
| MBRL 7 |  | - | + | + |  | I |
| MBRL 8 |  | + | + | - |  | XX |
| MBRL 9 |  | - | + | + |  | I |
| MBRL 10 |  | - | + | + |  | I |
| MBRL 11 |  | - | + | + |  | I |
| MBRL 12 |  | + | - | - |  | I |
| MBRL 13 |  | - | + | + |  | XII |
| MBRL 14 |  | - | + | - |  | XXII |
| MBRL 15 |  | + | + | - |  | XXII |
| MBRL 16 |  | + | + | - |  | III |
| MBRL 17 |  | - | - | + |  | XII |
| MBRL 18 |  | + | - | - |  | XXI |
| MBRL 19 |  | - | - | - |  | I |
| MBRL 20 |  | - | - | + |  | XII |
| MBRL 21 |  | - | + | + |  | I |
| MBRL 22 |  | - | + | + |  | III |
| MBRL 23 |  | - | + | - |  | I |
| MBRL 24 |  | + | + | + |  | III |
| MBRL 25 |  | + | - | + |  | XXII |
| MBRL 26 |  | + | + | + |  | VII |
| MBRL 27 |  | - | + | + |  | I |
| MBRL 28 |  | + | + | + |  | I |
| MBRL 29 |  | + | + | + |  | I |
| MBRL 30 |  | + | - | + |  | III |
| MBRL 31 |  | + | - | - |  | I |
| MBRL 32 |  | - | + | - |  | XV |
| MBRL 33 |  | + | - | - |  | III |
| MBRL 34 |  | - | + | - |  | XXIV |
| MBRL 35 |  | + | - | + |  | XII |
| MBRL 36 |  | - | - | + |  | XII |
| MBRL 37 |  | - | + | + |  | III |
| MBRL 38 |  | - | - | + |  | XII |
| MBRL 39 |  | + | + | + |  | I |
| MBRL 40 |  | - | - | + |  | XII |
| MBRL 41 |  | - | + | - |  | III |
| MBRL 42 |  | + | - | + |  | XXXI |
| MBRL 43 |  | - | + | + |  | I |
| MBRL 44 |  | - | + | + |  | I |
| MBRL 45 |  | - | + | - |  | III |
| MBRL 46 |  | - | - | - |  | XI |
| MBRL 47 |  | - | + | + |  | I |
| MBRL 48 |  | - | + | + |  | I |
| MBRL 49 |  | - | + | + |  | I |
| MBRL 50 |  | - | + | + |  | I |
| MBRL 51 |  | + | + | + |  | III |
| MBRL 52 |  | + | + | + |  | XXXI |
| MBRL 53 |  | - | - | - |  | XII |
| MBRL 54 |  | + | + | - |  | III |
| MBRL 55 |  | - | + | + |  | I |
| MBRL 56 |  | - | + | - |  | I |
| MBRL 57 |  | - | - | + |  | XII |
| MBRL 58 |  | - | - | + |  | XII |
| MBRL 59 |  | - | + | - |  | XXXI |
| MBRL 60 |  | - | + | + |  | I |
| MBRL 61 |  | - | - | - |  | XXII |
| MBRL 62 |  | + | + | + |  | XII |
| MBRL 63 |  | + | + | + |  | XXIII |
| MBRL 64 |  | - | + | - |  | XVI |
| MBRL 65 |  | + | + | + |  | XII |
| MBRL 66 |  | + | + | + |  | XII |
| MBRL 67 |  | - | + | + |  | II |
| MBRL 68 |  | + | + | - |  | XII |
| MBRL 69 |  | + | + | + |  | XII |
| MBRL 70 |  | - | + | + |  | XVII |
| MBRL 71 |  | - | + | + |  | I |
| MBRL 72 |  | + | + | - |  | XII |
| MBRL 73 |  | - | + | - |  | III |
| MBRL 74 |  | - | + | + |  | III |
| MBRL 75 |  | - | + | + |  | XVII |
| MBRL 76 |  | - | - | + |  | XIV |
| MBRL 77 |  | - | + | + |  | IV |
| MBRL 78 |  | - | + | + |  | III |
| MBRL 79 |  | - | + | + |  | XXVIII |
| MBRL 80 |  | + | + | - |  | III |
| MBRL 81 |  | - | - | - |  | III |
| MBRL 200 |  | + | - | + |  | III |
| MBRL 201 |  | - | + | + |  | I |
| MBRL 202 |  | + | + | + |  | III |
| MBRL 203 |  | + | + | + |  | III |
| MBRL 204 |  | - | - | - |  | XXV |
| MBRL 205 |  | - | - | - |  | XXV |
| MBRL 206 |  | + | + | + |  | V |
| MBRL 207 |  | + | + | + |  | V |
| MBRL 208 |  | - | + | - |  | XXV |
| MBRL 209 |  | - | - | - |  | XXV |
| MBRL 210 |  | - | - | - |  | XVIII |
| MBRL 211 |  | - | - | - |  | XV |
| MBRL 212 |  | - | + | - |  | XXV |
| MBRL 213 |  | + | + | + |  | IX |
| MBRL 214 |  | - | - | + |  | III |
| MBRL 215 |  | + | - | + |  | XXVII |
| MBRL 216 |  | - | - | + |  | I |
| MBRL 217 |  | + | - | + |  | XXV |
| MBRL 218 |  | - | - | - |  | XXV |
| MBRL 219 |  | - | - | - |  | X |
| MBRL 220 |  | - | + | - |  | XXVII |
| MBRL 221 |  | - | - | - |  | II |
| MBRL 222 |  | - | - | + |  | XIII |
| MBRL 223 |  | - | + | - |  | XXV |
| MBRL 224 |  | - | + | - |  | XXV |
| MBRL 225 |  | + | + | + |  | III |
| MBRL 226 |  | - | + | - |  | XXVI |
| MBRL 227 |  | + | - | + |  | VI |
| MBRL 228 |  | - | + | + |  | VI |
| MBRL 229 |  | - | - | - |  | XXV |
| MBRL 230 |  | - | - | - |  | XXIX |
| MBRL 231 |  | - | - | - |  | XXV |
| MBRL 232 |  | - | + | + |  | XXI |
| MBRL 233 |  | - | - | - |  | XXV |
| MBRL 234 |  | + | + | + |  | XII |
| MBRL 235 |  | - | + | - |  | XXX |
| MBRL 236 |  | - | + | - |  | XXX |
| MBRL 237 |  | - | + | - |  | XXVIII |
| MBRL 238 |  | + | + | + |  | III |
| MBRL 239 |  | + | + | - |  | XXV |
| MBRL 240 |  | + | - | - |  | XIX |
| MBRL 241 |  | - | + | - |  | VI |
| MBRL 242 |  | - | + | - |  | XXV |
| MBRL 243 |  | + | + | - |  | VIII |
| MBRL 244 |  | - | + | + |  | XXV |
| MBRL 245 |  | - | + | - |  | VI |
| MBRL 246 |  | - | + | - |  | XXV |
| MBRL 247 |  | - | + | + |  | XXV |
| MBRL 248 |  | - | + | + |  | III |
| MBRL 249 |  | - | + | - |  | XXV |
| MBRL 250 |  | - | + | - |  | III |
| MBRL 251 |  | + | + | + |  | III |
| MBRL 252 |  | - | + | - |  | III |
| MBRL 253 |  | - | - | - |  | XXII |
| MBRL 254 |  | + | + | + |  | XXII |
| MBRL 255 |  | - | + | - |  | XXII |
