# Supplementary material for: Actinobacterial diversity in limestone deposit sites in Hundung, Manipur (India) and their antimicrobial activities
Source: Front Microbiol. 2015 May 5;6:413. doi: 10.3389/fmicb.2015.00413 (PMC4419841; doi:10.3389/fmicb.2015.00413)

Supplementary Material

Actinobacterial diversity in limestone deposit sites in Hundung, Manipur (India) and their antimicrobial activities

Salam Nimaichand[1,2]*, Asem Mipeshwaree Devi[3], K. Tamreihao[1], Debananda S. Ningthoujam[1], Wen-Jun Li[2,4]*

[1]Microbial Biotechnology Research Laboratory, Department of Biochemistry, Manipur University, Canchipur, Imphal, Manipur, India
[2]State Key Laboratory of Biocontrol and Guangdong Key Laboratory of Plant Resources, School of Life Sciences, Sun Yat-Sen University, Guangzhou, China
[3]Molecular Genetics Laboratory, Department of Botany, North-Eastern Hill University, Shillong, Meghalaya, India
[4]Yunnan Institute of Microbiology, Yunnan University, Kunming, China

***Correspondence: Salam Nimaichand,** Department of Biochemistry, Manipur University, Canchipur, Imphal – 795003Manipur, India
Email: s.nimaichand@gmail.com

Wen-Jun Li
Email: liwenjun3@mail.sysu.edu.cn

Supplementary Figure S1 ARDRA-based dendrogram of the Hundung actinobacterial strains after digestion of amplified 16S rRNA gene product with the restriction enzymes *Hha*I and *Hin*fI. The dendrogram was generated using NTSYS software and are grouped with the UPGMA.

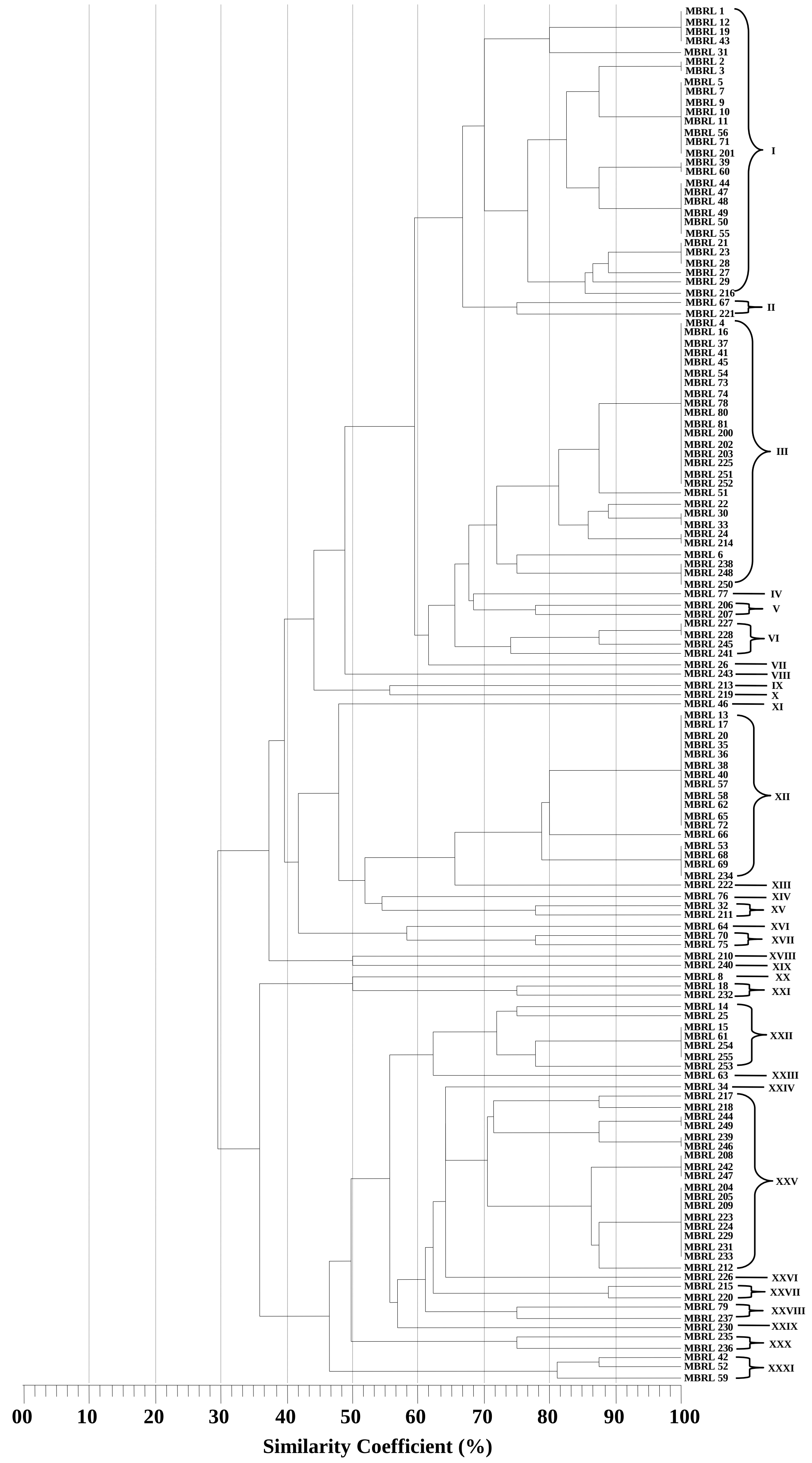

Supplement: Supplementary file 5 [file Image1.PDF]
